# Efficacy and safety of once-weekly insulin efsitora alfa versus daily basal insulin analogs in type 2 diabetes: a systematic review and meta-analysis

**DOI:** 10.1097/MS9.0000000000005075

**Published:** 2026-05-07

**Authors:** Bilal Wazir Khan, Marhaba Fatima, Rabia Amir, Rania Junejo, Shayan Nawaz, Gauhar Ullah, Ain Ul-Hayya, Arsalan Ahmed, Rutbaa Ayaz Shaikh, Imtiaz Ullah, Siraj Ud Din, Abdullah Qamar, Muhammad Shahreyar, Pratik Bhattarai

**Affiliations:** a Department of Medicine, Khyber Medical College, Peshawar, Khyber Pakhtunkhwa, Pakistan; b Department of Medicine, People’s University of Medical and Health Sciences for Women, Nawabshah, Sindh, Pakistan; c Department of Medicine, Jinnah Sindh Medical University, Karachi, Sindh, Pakistan; d Department of Medicine, Ziauddin Medical College, Karachi, Sindh, Pakistan; e Department of Medicine, University College of Medicine and Dentistry, Lahore, Punjab, Pakistan; f Department of Medicine, Ayub Medical College, Abbottabad, Khyber Pakhtunkhwa, Pakistan; g Department of Medicine, Liaquat University of Medical Health Sciences, Jamshoro, Sindh, Pakistan; h Department of Medicine, Dow Medical College, Karachi, Sindh, Pakistan; i Department of Medicine, Aziz Fatimah Medical College, Faisalabad, Punjab, Pakistan; j Department of Medicine, Shifa College of Medicine, Islamabad, Pakistan; k Department of Medicine, Manipal College of Medical Sciences, Pokhara, Nepal

**Keywords:** basal insulin, insulin efsitora alfa, once-weekly insulin, systematic review and meta-analysis, type 2 diabetes mellitus

## Abstract

**Background:**

Type 2 diabetes mellitus (T2DM) often progresses to require basal insulin therapy. Despite the efficacy of once-daily basal insulins such as glargine and degludec, daily injections remain a barrier to adherence. Insulin efsitora alfa, a novel once-weekly fragment crystallizable (Fc)-fusion basal insulin, may improve treatment convenience while maintaining glycemic control.

**Objective:**

To evaluate the efficacy and safety of once-weekly insulin efsitora alfa compared with once-daily basal insulin analogs in adults with T2DM.

**Methods:**

An updated systematic review and meta-analysis was conducted according to PRISMA 2020 guidelines and registered in PROSPERO (CRD420251118205). PubMed, Embase, and CENTRAL were searched up to 30 July 2025 for randomized controlled trials (RCTs) comparing insulin efsitora alfa with once-daily basal insulins. Pooled analyses were performed using a random-effects model in RevMan 5.4. Risk of bias was assessed with the Cochrane RoB-2 tool, and evidence certainty with the Grading of Recommendations, Assessment, Development, and Evaluation (GRADE) approach.

**Results:**

Six RCTs comprising 4115 participants were included. Once-weekly insulin efsitora showed non-inferior HbA1c reduction compared with daily basal insulin (mean difference = −0.04; 95% confidence interval: –0.13 to 0.05; *P* = 0.35). No significant differences were observed in fasting serum glucose or body weight change. Incidents of level 1, level 2 (clinically significant), and level 3 (severe) hypoglycemia, as well as adverse and serious adverse events, were comparable between groups.

**Conclusion:**

Once-weekly insulin efsitora alfa demonstrates comparable efficacy and safety to daily basal insulin. However, further large-scale, long-term trials are warranted to confirm its durability, safety, and real-world effectiveness.

## Introduction

Type 2 diabetes mellitus (T2DM) accounts for the majority of diabetes cases globally, affecting an estimated 529 million people worldwide in 2021, and is projected to exceed 1.31 billion by 2050^[^1^]^. It is a progressive metabolic disorder characterized by insulin resistance and β-cell dysfunction, leading to chronic hyperglycemia and the need for pharmacologic intervention as the disease advances^[^2^]^. In its early stages, T2DM is typically managed with lifestyle modifications and oral hypoglycemic agents, such as metformin, sulfonylureas, or SGLT2 inhibitors^[^3^]^. However, a significant proportion of patients eventually require basal insulin therapy to maintain adequate glycemic control^[^4^]^.

Long-acting basal insulin analogs, including insulin glargine and insulin degludec, have been widely used for their once-daily dosing convenience and reduced risk of hypoglycemia compared to traditional insulins^[^5,6^]^. Nevertheless, daily injections remain a barrier to adherence for many patients, contributing to treatment inertia and suboptimal outcomes^[^7^]^. In response, novel once-weekly basal insulins such as insulin icodec and insulin efsitora alfa (Fc-fusion insulin) have been developed, aiming to reduce injection burden while maintaining efficacy.

Recent phase 3 trials have demonstrated the non-inferiority of once-weekly basal insulin regimens compared to daily basal insulin in terms of glycemic control, with comparable safety profiles^[^8–10^]^. Moreover, patient-reported outcomes suggest potential benefits in treatment satisfaction and adherence with reduced injection frequency^[^11^]^. However, the emerging evidence remains fragmented across various trials due to differences in study populations and insulin type. We specifically focus here on type 2 diabetes patients and insulin efsitora to ensure consistency across studies^[^12,13^]^.

We aim to synthesize the current clinical evidence comparing once-weekly insulin efsitora alpha with once-daily basal insulin regimens in adults with T2DM. Our objective is to evaluate efficacy based on change in HbA1c, fasting blood glucose, body weight, and safety based on adverse events, hypoglycemia, and death.

## Methods

Our systematic review and meta-analysis was reported following the Preferred Reporting Items for Systematic Reviews and Meta-Analysis (PRISMA) 2020^[^14^]^ guidelines and registered on PROSPERO (Reg no: CRD420251118205). No ethical approval was required for our study.

### Search strategy

PubMed, CENTRAL – Cochrane Central Register of Controlled Trials, and Embase were used for the search strategy. These databases were searched from inception to 30 July 2025 with a filter of English language. The comprehensive search strategy included Medical Subject Headings terms and keywords like “Type 2 diabetes,” “Type 2 diabetes mellitus,” “Efsitora,” “Insulin Efsitora alfa,” “Basal Insulin,” “Degludec,” and “Insulin Degludec.” The detailed search strategy used for each database is available in the Supplemental Digital Content Appendix, available at: http://links.lww.com/MS9/B192. The reference lists of related articles were manually searched to retrieve additional articles.

### Study selection and eligibility criteria

Two authors (A.A. and A.H.) independently screened the articles through Rayyan, with disagreements being resolved by a third author (M.F.). The screening was started with the title and abstract-level screening, followed by full-text review. The eligibility criteria were (1) population: patients with T2DM, (2) intervention: efsitora Alfa, (3) control: once-daily insulin analogs such as insulin glargine, insulin degludic, and (4) study design: randomized controlled trials. All other types of studies, like letters, case reports, and systematic reviews, were excluded. Patients with type 1 diabetes, without diabetes, and using insulin Icodec as an intervention were excluded.

### Data extraction and outcomes

Data were extracted by two authors (G.U. and B.K.) independently using Google Sheets, and a third author (M.F.) resolved all the disagreements. Study characteristics data were study ID, registration number, country, study design, frequency, sample size, and follow-up period. The patients’ characteristics data were age, Body Mass Index, duration of diabetes, HbA1c%, and fasting serum glucose level. Our efficacy outcomes were change in HbA1c (%) from baseline, change in fasting serum glucose, body weight change from baseline, and our safety outcomes were incidence of hypoglycemia, death, and adverse events [any adverse events, serious adverse events, treatment-emergent adverse events (TEAEs)].

HIGHLIGHTS

- Once-weekly insulin efsitora alfa achieved non-inferior HbA1c reduction compared with once-daily basal insulin in adults with type 2 diabetes.
- Fasting blood glucose and body weight changes were comparable between weekly efsitora and daily basal insulin analogs.
- Overall hypoglycemia risk (levels 1–3) did not differ significantly between treatment regimens.
- Once-weekly insulin efsitora was associated with a significantly lower risk of nocturnal hypoglycemia.
- Treatment-emergent adverse events occurred slightly more frequently with efsitora, while serious adverse events and mortality were similar.
- Weekly basal insulin may reduce injection burden without compromising glycemic efficacy or safety.

### Quality assessment

For quality assessments of the studies, the Cochrane Risk of Bias 2 (ROB-2) tool was used. All the studies were comprehensively screened and were rated as low risk, moderate risk, and high risk of bias. The quality of studies was assessed by two authors (S.D. and I.U.) independently, and the third author (M.F.) resolved any discrepancies between the two authors.

### GRADE assessment

The certainty of evidence in this meta-analysis was assessed using the Grading of Recommendations, Assessment, Development, and Evaluation (GRADE) approach. Two independent reviewers evaluated the evidence and categorized it as high, moderate, low, or very low certainty^[^15^]^. Any disagreements were resolved through discussion and mutual consensus.

### Data analysis

All the data were analyzed using RevMan 5.4. For dichotomous outcomes, we calculated risk ratios with a 95% confidence interval (CI) using the random-effect model. Continuous outcomes were analyzed using generic inverse variation. The 90% CI and 95% CI were converted into standard error using the RevMan calculator. We calculated the mean difference (MD) with CI. The studies that have *P*-values less than or equal to 0.05 were considered statistically significant for all outcomes. Heterogeneity was assessed using *I*^2^ statistics. *I*^2^ values of 25%, > 50%, and >75% indicate low, intermediate, and considerable degrees of heterogeneity, respectively. In case of statistical heterogeneity, we have used subgroup and sensitivity analyses. One subgroup was based on insulin-naïve and insulin-experienced populations, and the other was based on the control group, such as insulin glargine and insulin degludec. In case of any residual heterogeneity, a meta-regression using metafor package in R (4.4.3) with RStudio was done. Moderators used were loading dose of insulin, titration frequency, and dose difference between insulin efsitora and basal insulin (glargine and degludec). Loading dose was coded as a binary variable (yes/no), indicating whether an initial loading dose of insulin was administered. Titration frequency was coded as a categorical variable reflecting the protocol-defined frequency of dose adjustments during the study period. Dose difference was coded as a continuous variable, calculated as the difference between the weekly equivalent dose of insulin efsitora and the daily dose of comparator basal insulin (glargine or degludec).

## Results

### Study selection

**Figure 1.**
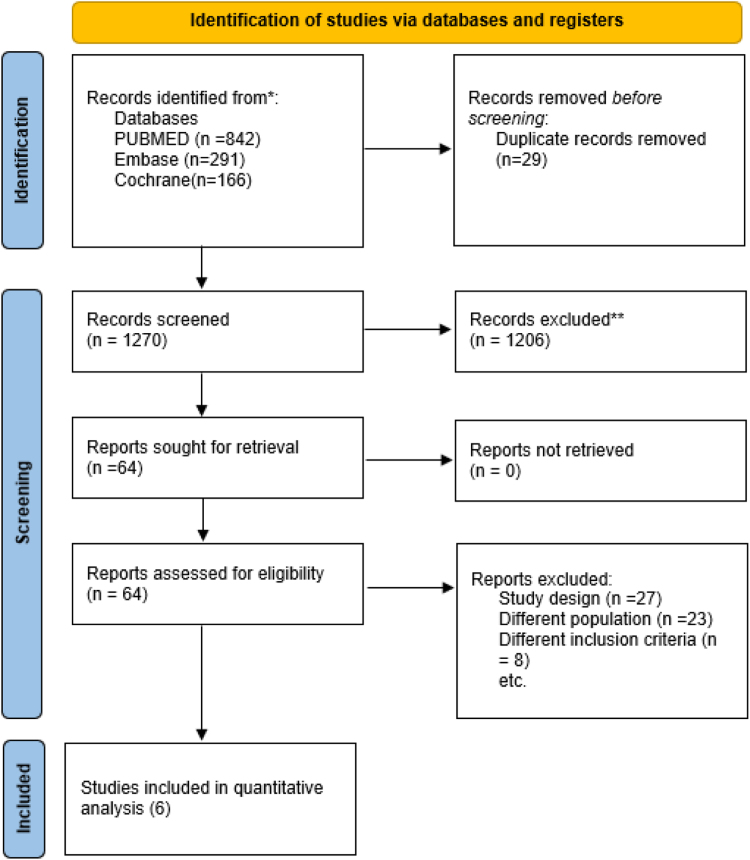
PRISMA 2020 flow diagram, which included searches of databases and registers only.

The electronic database search (PubMed, Embase, Cochrane CENTRAL) yielded 1299 records. After the removal of 29 duplicates, 1270 records were screened by title and abstract. A total of 64 full-text articles were assessed for eligibility, of which six randomized controlled trials (RCTs)^[^13,16–20^]^ met the prespecified inclusion criteria (Fig. 1).

### Baseline characteristics

The six included RCTs were published between 2023 and 2025 across North America, Europe, and Asia, and together enrolled 4115 adults with T2DM (2292 randomized to once-weekly insulin efsitora; 1823 to once-daily basal insulin analogs). Follow-up duration ranged from 26 to 52 weeks, corresponding to the primary efficacy assessment time points. Participants were typically middle-aged (mean [SD]: 58.9 [9.1] years), with baseline HbA1c levels 7.8–8.4%. Most were inadequately controlled on oral agents, with or without prior insulin exposure. Comparator basal insulins included insulin glargine and insulin degludec. Baseline characteristics of patients are summarized in Table 1, and Supplemental Digital Content Table S1, available at: http://links.lww.com/MS9/B192.

**Table 1:** Baseline characteristics of patients included in the analysis.

Study ID	Age (years) Mean SD		BMI (kg/m^2^) Mean SD		Duration of diabetes (years) Mean SD		HbA1c or glycated hemoglobin (%) Mean SD		Fasting serum glucose or FSG (mg/dl) Mean (SD)	
	Intervention	Control	Intervention	Control	Intervention	Control	Intervention	Control	Intervention	Control
Blevins *et al*^[17]^	58 3 (10 5)	59 4 (10 5)	31 85 (5 48)	31 84 (5 48)	16 6 (8 8)	16 9 (9 0)	8 36 (0 78)	8 36 (0 80)	148 3(49.4)	152 9 (54 7)
Bue-Valleskey *et al*^[19]^	57.3 (9.7)	59.4 (9.1)	32.3 (5.4)	31.6 (5.5)	10.4 (6.8)	9.7 (6.0)	8.1 (0.8)	8.0 (0.8)	170.2 (42.0)	160.7 (36.7)
Frias *et al*^[16]^	59 9 (10 6)	60 8 (10 0)	32 5 (5 9)	31 8 (5 7)	14 6 (8 8)	15 1 (8 0)	8 1 (0 9)	8 1 (0 9)	141 2 (50 2)	144 5 (51 0)
Philis-Tsimikas *et al*^[13]^	62.0 (10.4)	61.0 (9.68)	29 9 (5.72)	30.33 (5.81)	15.0 (8.54)	14.7 (7.08)	NR	NR	131.4 (41.23)	131 67 (38.73)
Rosenstock *et al*^[18]^	56.4 (10.0)	56.2 (9.7)	32.5 (5.8)	31.3 (6.1)	9.2 (6.6)	9.6 (6.9)	8.2 (0.91)	8.27 (1.07)	161 (48)	161 (52)
Wysham *et al*^[20]^	57.6(10.6)	57.3 (11.0)	30.44 (5.85)	30.72 (5.90)	11.78 (7.54)	11.42 (6.97)	8.21 (0.96)	8.23 (0.96)	162.32 (45.79)	165.13 (48.78)

NR, not reported.

### Assessment of bias

Risk of bias was assessed using the Cochrane RoB-2.0 tool. All six RCTs were at low risk of bias in randomization, outcome assessment, and missing data, but all raised some concerns for deviations from intended interventions (Supplemental Digital Content Figures S1 and S2, available at: http://links.lww.com/MS9/B192). Funnel plot and Egger’s regression were not performed due to fewer than 10 trials per outcome, precluding reliable assessment of publication bias.

### GRADE assessment

Using the GRADEpro Guideline Development Tool, the GRADE framework was used to assess the certainty of evidence across all outcomes. The overall quality ranged from high to low. High-certainty evidence was found for primary analyses of body weight, combined level 2 and 3 hypoglycemia, level 3 hypoglycemia, nocturnal hypoglycemia, mortality, hypersensitivity, injection site reactions, medication errors, serious adverse events, and TEAEs. Meanwhile, moderate-certainty evidence supported primary analyses of glycated hemoglobin (HbA1c), fasting blood glucose, any adverse event, level 1 hypoglycemia, and level 2 hypoglycemia. Downgrading was mainly due to methodological issues (e.g., lack of blinding), inconsistency, and imprecision from small sample sizes and wide CIs. Detailed GRADE assessment (including that of subgroup and sensitivity analyses) has been provided in Supplemental Digital Content Table S2, available at: http://links.lww.com/MS9/B192.

### Meta-analysis of efficacy outcomes

#### HbA1c (%)

**Figure 2.**
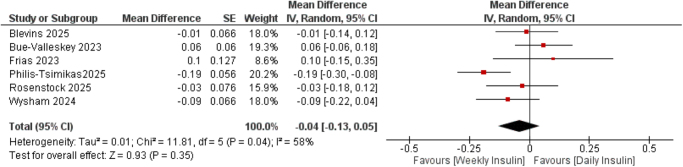
Forest plot of mean difference in HbA1C (%) comparing once-weekly insulin efsitora vs once-daily basal insulin analogs (glargine and degludec).

Six studies^[^13,16–20^]^ with a combined sample size of 4115 participants evaluated the change in HbA1c. The pooled analysis showed no significant difference between once-weekly insulin efsitora and once-daily basal insulin analogs (MD = −0.04, 95% CI: −0.13 to 0.05; *P* = 0.35) (Fig. 2).

Moderate heterogeneity was observed (*I*^2^ = 58%, τ^2^ = 0.01), which reduced to 0% after excluding the Philis trial^[^13^]^. Leave-one-out sensitivity analyses confirmed the robustness of findings, as no single study significantly altered the overall effect size or its non-significance (all *P* > 0.80) (Supplemental Digital Content Figure S3, available at: http://links.lww.com/MS9/B192).

Subgroup analyses by prior insulin exposure in insulin-naïve (MD = −0.02, 95% CI: −0.11 to 0.07; *P* = 0.67) vs insulin-experienced participants (MD = −0.06, 95% CI: −0.22 to 0.10; *P* = 0.49) revealed no statistical significance (Supplemental Digital Content Figure S4, available at: http://links.lww.com/MS9/B192).

Subgroup by comparator type in glargine (MD = −0.08, 95% CI: −0.20 to 0.04; *P* = 0.18) vs degludec (MD = 0.00, 95% CI: −0.11 to 0.12; *P* = 0.93) did not reveal significant effect modification (Supplemental Digital Content Figure S5, available at: http://links.lww.com/MS9/B192).

#### Fasting blood glucose

**Figure 3.**
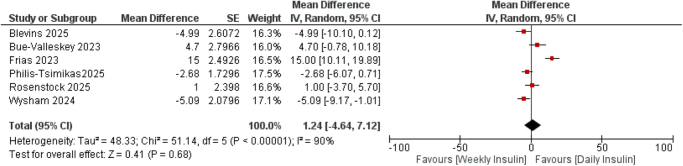
Forest plot of mean difference of Fasting Blood Glucose (mg/dl) comparing once-weekly insulin efsitora versus once-daily basal insulin analogs (glargine and degludec).

Six studies^[^13,16–20^]^ reported data for fasting blood glucose (mg/dl), with a sample size of 4115. The pooled estimate showed no significant difference between weekly and daily insulin regimens (MD = 1.24, 95% CI: −4.64 to 7.12; *P* = 0.68) (Fig. 3).

Marked heterogeneity was detected (*I*^2^ = 90%) (Fig), which reduced to 64%, (Fig), after removing Frias^[^16^]^ (Supplemental Digital Content Figure S6, available at: http://links.lww.com/MS9/B192).

Subgroup analyses by prior insulin exposure in insulin-naïve (MD = −0.02, 95% CI: −5.71 to 5.67; *P* = 0.99) vs insulin-experienced participants (MD = 2.40, 95% CI: −9.19 to 14.00; *P* = 0.68) revealed no statistical significance (Supplemental Digital Content Figure S7, available at: http://links.lww.com/MS9/B192).

Subgroup by comparator type in glargine (MD = −2.20, 95% CI: −5.25 to 0.85; *P* = 0.16) vs degludec (MD = 4.81, 95% CI: −7.32 to 16.95; *P* = 0.44) did not reveal significant effect modification (Supplemental Digital Content Figure S8, available at: http://links.lww.com/MS9/B192).

Meta-regression demonstrated that dose difference was a statistically significant moderator of the treatment effect on FBG (QM [model Q statistic testing moderators] = 12.23, *P* = 0.0005). The regression coefficient was −0.38 (95% CI: −0.59 to −0.17), (Supplemental Digital Content Table S3, available at: http://links.lww.com/MS9/B192) indicating that greater dose differences between treatment arms were associated with a larger reduction in fasting blood glucose. This accounted for a substantial proportion of between-study heterogeneity (*R*^2^ = 77.5%). However, residual heterogeneity remained significant (QE [residual Q statistic for unexplained heterogeneity] = 11.40, *P* = 0.022), with an *I*^2^ of 65.8%, suggesting that additional unmeasured factors may contribute to the observed variability. In contrast, meta-regression showed that neither titration frequency nor the loading dose significantly modified the effect of treatment on fasting blood glucose. Both moderators explained none of the between-study heterogeneity (*R*^2^ = 0%), (Supplemental Digital Content Table S3, available at: http://links.lww.com/MS9/B192) .

#### Body weight

**Figure 4.**
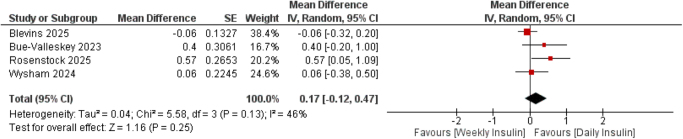
Forest plot of mean difference of Body Weight (kg) comparing once-weekly insulin efsitora versus once-daily basal insulin analogs (glargine and degludec).

Four trials^[^17–20^]^ with a total of 2731 participants assessed changes in body weight (kg). The pooled analysis showed no significant difference between once-weekly insulin efsitora and once-daily basal insulin analogs (MD = 0.17 kg, 95% CI: −0.12 to 0.47; *P* = 0.25) (Fig. 4).

Moderate heterogeneity was detected (*I*^2^ = 46%), which reduced to 0% after excluding the Rosenstock trial^[^18^]^(Supplemental Digital Content Figure S9, available at: http://links.lww.com/MS9/B192). Subgroup analyses did not demonstrate evidence of effect modification (*P* for interaction = 0.25).

Subgroup analyses by prior insulin exposure showed borderline significance in insulin-naive patients (MD = 0.31, 95% CI: −0.01 to 0.62; *P* = 0.05) and no significant difference in insulin-experienced participants (MD = −0.06, 95% CI: −0.32 to 0.20; *P* = 0.25) (Supplemental Digital Content Figure S10, available at: http://links.lww.com/MS9/B192).

Subgroup by comparator type in glargine (MD = 0.21, 95% CI: −0.40 to 0.83; *P* = 0.49) vs degludec (MD = 0.18, 95% CI: −0.18 to 0.53; *P* = 0.32) did not reveal significant effect modification (Supplemental Digital Content Figure S11, available at: http://links.lww.com/MS9/B192).

### Safety outcomes

#### Any adverse event

**Figure 5.**
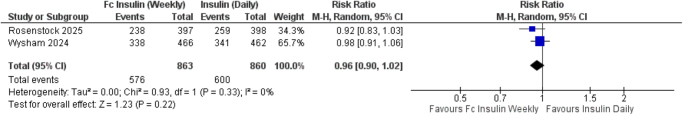
Forest plot of any adverse events comparing once-weekly insulin efsitora versus once-daily basal insulin analogs (glargine and degludec).

Two studies^[^18,20^]^ reported data for any adverse event, with a sample size of 1723 (863 in weekly insulin and 860 in daily insulin). The pooled analysis showed no statistically significant difference between the two groups (RR [risk ratio] = 0.96, 95% CI: 0.90–1.02, *P* = 0.22), with low heterogeneity (*I*^2^ = 0%) (Fig. 5).

#### Serious adverse event

Six studies^[^13,16–20^]^ reported serious adverse events, including a total of 4115 participants. The pooled analysis showed no statistically significant difference between once-weekly insulin efsitora and once-daily basal insulin analogs (RR = 1.19, 95% CI: 0.97–1.47; *P* = 0.10). Reported heterogeneity was low (*I*^2^ = 0%) (Supplemental Digital Content Figure S38, available at: http://links.lww.com/MS9/B192).

Subgroup analyses by prior insulin exposure in insulin-naïve (RR = 1.16, 95% CI: 0.84–1.61; *P* = 0.37) vs insulin-experienced participants (RR = 1.19, 95% CI: 0.87–1.63; *P* = 0.29) revealed no statistical significance (Supplemental Digital Content Figure S12, available at: http://links.lww.com/MS9/B192).

Subgroup by comparator type in glargine (RR = 1.29, 95% CI 0.99–1.68; *P* = 0.06) vs degludec (RR = 1.03, 95% CI: 0.72–1.47; *P* = 0.87) did not reveal significant effect modification (Supplemental Digital Content Figure S13, available at: http://links.lww.com/MS9/B192).

#### Treatment-emergent adverse events

Four studies^[^13,16,17,19^]^ including 2392 participants reported TEAEs. The pooled analysis showed a higher incidence with once-weekly insulin efsitora compared with once-daily basal insulin (RR = 1.11, 95% CI: 1.04–1.18; *P* = 0.003). No heterogeneity was detected (*I*^2^ = 0%) (Supplemental Digital Content Figure S39, available at: http://links.lww.com/MS9/B192).

Subgroup analyses by prior insulin exposure showed no significant effect among insulin-naïve participants (RR = 1.10, 95% CI: 0.86–1.42; *P* = 0.45), whereas a statistically significant benefit was observed in insulin-experienced participants (RR = 1.11, 95% CI: 1.03–1.19; *P* = 0.003) (Supplemental Digital Content Figure S14, available at: http://links.lww.com/MS9/B192).

Subgroup analyses by comparator type showed a statistically significant effect with glargine (RR = 1.10, 95% CI: 1.01–1.20; *P* = 0.03), whereas no significant effect was observed with degludec (RR = 1.11, 95% CI: 0.96–1.28; *P* = 0.16) (Supplemental Digital Content Figure S15, available at: http://links.lww.com/MS9/B192).

#### Hypersensitivity events

Six studies^[^13,16–20^]^ reported hypersensitivity events in 4115 participants. The pooled analysis demonstrated no statistically significant difference between treatment groups (RR = 1.36, 95% CI: 0.88–2.12; *P* = 0.17) (Supplemental Digital Content Figure S40, available at: http://links.lww.com/MS9/B192). Reported heterogeneity was 35%, which reduced to 22% after excluding Rosenstock^[^18^]^ (Supplemental Digital Content Figure S16, available at: http://links.lww.com/MS9/B192).

Subgroup analyses by prior insulin exposure in insulin-naïve (RR = 1.23, 95% CI: 0.48–3.14; *P* = 0.67) vs insulin-experienced participants (RR = 1.58, 95% CI: 0.84–2.96; *P* = 0.16) revealed no statistical significance (Supplemental Digital Content Figure S17, available at: http://links.lww.com/MS9/B192).

Subgroup by comparator type in glargine (RR = 1.15, 95% CI: 0.56–2.33; *P* = 0.70) vs degludec (RR = 1.79, 95% CI: 0.84–3.81; *P*= 0.13) did not reveal significant effect modification (Supplemental Digital Content Figure S18, available at: http://links.lww.com/MS9/B192).

Leave-one-out sensitivity analyses confirmed robustness (overall *P* = 0.06).

#### Injection site reaction/adverse event

Six studies^[^13,16–20^]^ with 4115 participants reported injection-site reactions. The pooled analysis showed no significant difference between treatment groups (RR = 1.31, 95% CI: 0.80–2.14; *P* = 0.29). No heterogeneity was detected (*I*^2^ = 0%) (Supplemental Digital Content Figure S41, available at: http://links.lww.com/MS9/B192).

Subgroup analyses by prior insulin exposure in insulin-naïve (RR = 1.26, 95% CI: 0.68–2.32; *P* = 0.46) vs insulin-experienced participants (RR = 1.41, 95% CI: 0.61–3.25; *P* = 0.42) revealed no statistical significance (Supplemental Digital Content Figure S19, available at: http://links.lww.com/MS9/B192).

Subgroup by comparator type in glargine (RR = 1.23, 95% CI: 0.61–2.46; *P* = 0.56) vs degludec (RR = 1.39, 95% CI: 0.69–2.80; *P* = 0.35) did not reveal significant effect modification (Supplemental Digital Content Figure S20, available at: http://links.lww.com/MS9/B192).

#### Level 1 hypoglycemia

Six studies^[^13,16–20^]^ with 4115 participants reported level 1 hypoglycemia. Pooled results indicated no significant between-group difference (RR = 1.06, 95% CI: 0.98–1.16; *P* = 0.16). Reported heterogeneity was *I*^2^ = 71% (Supplemental Digital Content Figure S42, available at: http://links.lww.com/MS9/B192), which reduced to *I*^2^ = 58% after excluding Wysham^[^20^]^ (Supplemental Digital Content Figure S21, available at: http://links.lww.com/MS9/B192).

Subgroup analyses by prior insulin exposure in insulin-naïve (RR = 1.10, 95% CI: 0.89–1.37; *P* = 0.37) vs insulin-experienced participants (RR = 1.04, 95% CI: 0.96–1.12; *P* = 0.37) revealed no statistical significance (Supplemental Digital Content Figure S22, available at: http://links.lww.com/MS9/B192).

Subgroup analysis by comparator showed no difference with glargine (RR = 1.00, 95% CI: 0.94–1.07; *I*^2^ = 52%), but a higher risk with degludec (RR = 1.21, 95% CI: 1.10–1.33; *I*^2^ = 0%). The between-subgroup difference was significant (χ^2^ = 10.07, *P* = 0.002), indicating the comparator type is a source of heterogeneity. (Supplemental Digital Content Figure S23, available at: http://links.lww.com/MS9/B192).

Leave-one-out sensitivity analyses confirmed robustness (overall *P* = 0.52). Heterogeneity decreased from *I*^2^ = 71% to *I*^2^ = 58% after excluding Wysham.

#### Level 2 hypoglycemia

Five studies^[^13,16,18–20^]^ with 3385 participants reported level 2 hypoglycemia. Pooled analysis showed no significant difference between groups (RR = 1.04, 95% CI: 0.87–1.25; *P* = 0.67). Reported heterogeneity was *I*^2^ = 65% (Supplemental Digital Content Figure S43, available at: http://links.lww.com/MS9/B192), which reduced to *I*^2^ = 45% after excluding Wysham^[^20^]^ (Supplemental Digital Content Figure S24, available at: http://links.lww.com/MS9/B192).

Subgroup analyses by prior insulin exposure in insulin-naïve (RR = 1.13, 95% CI: 0.77–1.65; *P* = 0.52) vs insulin-experienced participants (RR = 0.99, 95% CI: 0.78–1.24; *P* = 0.90) revealed no statistical significance (Supplemental Digital Content Figure S25, available at: http://links.lww.com/MS9/B192).

Subgroup by comparator type in glargine (RR = 0.99, 95% CI: 0.79–1.24; *P* = 0.96) vs degludec (RR = 1.13, 95% CI: 0.76–1.69; *P* = 0.54) did not reveal significant effect modification (Supplemental Digital Content Figure S26, available at: http://links.lww.com/MS9/B192)

Leave-one-out sensitivity analyses confirmed robustness (overall *P* = 0.68). Heterogeneity decreased from *I*^2^ = 65% to *I*^2^ = 45% after excluding Wysham.

#### Level 3 hypoglycemia

Six studies^[^13,16–20^]^ with 4115 participants reported level 3 hypoglycemia. Meta-analysis demonstrated no significant difference between groups (RR = 0.97, 95% CI: 0.42–2.24; *P* = 0.94). No heterogeneity was detected (*I*^2^ = 0%) (Supplemental Digital Content Figure S44, available at: http://links.lww.com/MS9/B192).

Subgroup analyses by prior insulin exposure in insulin-naïve (RR = 0.28, 95% CI: 0.02–3.94; *P* = 0.35) vs insulin-experienced participants (RR = 1.25, 95% CI: 0.50–3.17; *P* = 0.63) revealed no statistical significance (Supplemental Digital Content Figure S27, available at: http://links.lww.com/MS9/B192).

Subgroup by comparator type in glargine (RR = 1.15, 95% CI: 0.46–2.88; *P* = 0.77) vs degludec (RR = 0.42, 95% CI: 0.01–13.41; *P* = 0.62) did not reveal significant effect modification (Supplemental Digital Content Figure S28, available at: http://links.lww.com/MS9/B192).

#### Combined level 2 and 3 hypoglycemia

Six studies^[^13,16–20^]^ with 4115 participants reported combined level 2 and 3 hypoglycemia. The pooled estimate showed no significant difference between groups (RR = 1.02, 95% CI: 0.90–1.15; *P* = 0.76). Heterogeneity was decreased from *I*^2^ = 48% (Supplemental Digital Content Figure S45, available at: http://links.lww.com/MS9/B192) to *I*^2^ = 25% after excluding Wysham^[^20^]^ (Supplemental Digital Content Figure S29, available at: http://links.lww.com/MS9/B192).

Subgroup analyses by prior insulin exposure in insulin-naïve (RR = 1.09, 95% CI: 0.77–1.55; *P* = 0.61) vs insulin-experienced participants (RR = 1.00, 95% CI: 0.90–1.11; *P* = 0.98) revealed no statistical significance (Supplemental Digital Content Figure S30, available at: http://links.lww.com/MS9/B192).

Subgroup by comparator type in glargine (RR = 1.00, 95% CI: 0.89–1.12; *P* = 0.98) vs degludec (RR = 1.10, 95% CI: 0.78–1.55; *P* = 0.59) did not reveal significant effect modification (Supplemental Digital Content Figure S31, available at: http://links.lww.com/MS9/B192).

Leave-one-out sensitivity analyses confirmed robustness (overall *P* = 0.70).

#### Nocturnal hypoglycemia

Six studies^[^13,16–20^]^ with 4115 participants reported nocturnal hypoglycemia. Pooled analysis showed significantly fewer events with once-weekly insulin efsitora (RR = 0.79, 95% CI: 0.67–0.95; *P* = 0.01). No heterogeneity was detected (*I*^2^ = 0%) (Supplemental Digital Content Figure S46, available at: http://links.lww.com/MS9/B192).

Subgroup analyses by prior insulin exposure showed no significant effect among insulin-naïve participants (RR = 0.84, 95% CI: 0.55–1.28; *P* = 0.42), whereas a statistically significant benefit was observed in insulin-experienced participants (RR = 0.85, 95% CI: 0.75–0.96; *P* = 0.009) (Supplemental Digital Content Figure S32, available at: http://links.lww.com/MS9/B192).

Subgroup analyses by comparator type showed a statistically significant effect with glargine (RR = 0.87, 95% CI: 0.77–0.98; *P* = 0.02), whereas no significant effect was observed with degludec (RR = 0.81, 95% CI: 0.62–1.05; *P* = 0.12) (Supplemental Digital Content Figure S33, available at: http://links.lww.com/MS9/B192).

#### Death

Five studies^[^13,17–20^]^ with 3717 participants reported death as an outcome. The pooled analysis showed no significant difference between groups (RR = 1.22, 95% CI: 0.49–3.03; *P* = 0.67). No heterogeneity was detected (*I*^2^ = 0%) (Supplemental Digital Content Figure S47, available at: http://links.lww.com/MS9/B192).

Subgroup analyses by prior insulin exposure in insulin-naïve (RR = 1.01, 95% CI: 0.30–3.44; *P* = 0.99) vs insulin-experienced participants (RR = 1.54, 95% CI: 0.39–6.02; *P* = 0.53) revealed no statistical significance (Supplemental Digital Content Figure S34, available at: http://links.lww.com/MS9/B192).

Subgroup by comparator type in glargine (RR = 1.28, 95% CI: 0.46–3.62; *P* = 0.64) vs degludec (RR = 1.02, 95% CI: 0.15–6.94; *P* = 0.98) did not reveal significant effect modification (Supplemental Digital Content Figure S35, available at: http://links.lww.com/MS9/B192).

#### Medication error

Four studies^[^13,17,18,20^]^ with 3504 participants reported medication errors. The pooled analysis showed no statistically significant difference (RR = 2.44, 95% CI: 0.78–7.62; *P* = 0.12). No heterogeneity was detected (*I*^2^ = 0%) (Supplemental Digital Content Figure S48, available at: http://links.lww.com/MS9/B192).

Subgroup analyses by prior insulin exposure in insulin-naïve (RR = 1.53, 95% CI: 0.40–5.92; *P* = 0.54) vs insulin-experienced participants (RR = 3.92, 95% CI: 0.21–72.79; *P* = 0.36) revealed no statistical significance (Supplemental Digital Content Figure S36, available at: http://links.lww.com/MS9/B192).

Subgroup by comparator type in glargine (RR = 3.61, 95% CI: 0.61–21.33; *P* = 0.16) vs degludec (RR = 1.32, 95% CI: 0.30–5.87; *P* = 0.71) did not reveal significant effect modification (Supplemental Digital Content Figure S37, available at: http://links.lww.com/MS9/B192).

## Discussion

We evaluated the efficacy and safety of once-weekly insulin efsitora with once-daily basal insulin in adults with type 2 diabetes. Our analysis showed no significant difference in HbA1c, fasting blood glucose, or body weight between once-weekly efsitora and once-daily basal insulin. Safety outcomes were mostly comparable between the two groups except for TEAEs and nocturnal hypoglycemia. Once-weekly efsitora was associated with a higher risk of TEAEs and a lower risk of nocturnal hypoglycemia. Regarding subgroup effects, they were broadly consistent across most efficacy and safety outcomes except for TEAEs, level 1 hypoglycemia, and nocturnal hypoglycemia.

HbA1c reduction was comparable between regimens, consistent with previous meta-analyses such as Dutta, which demonstrated weekly insulin efsitora’s efficacy and safety compared with insulin glargine or degludec^[^21^]^. Importantly, subgroup analyses for efficacy outcomes did not identify differential effects among insulin-naïve vs insulin-experienced participants, or between comparator analogs, underscoring the consistency of the treatment effect.

The lack of significant differences in fasting plasma glucose and body weight further supports similar therapeutic performance between regimens. However, heterogeneity was notable for fasting glucose outcomes. Importantly, meta-regression identified dose difference between treatment arms as a statistically significant and quantitatively important moderator of fasting blood glucose outcomes, explaining a large proportion of between-study heterogeneity (*I*^2^ = 77.5%). It was noted that Frias^[^16^]^ titrated each arm to different fasting plasma glucose targets, with degludec’s target being substantially lower at ≤5·6 mmol/L, compared to basal insulin-focused (BIF)-A1 at ≤7·8 mmol/L or BIF-A2 at ≤6·7 mmol/L. These asymmetric glycemic targets likely produced meaningful dose imbalances between study arms, which is consistent with the meta-regression finding that larger dose differences were associated with greater reductions in fasting blood glucose. After a leave-out analysis, it was found that without Frias^[^16^]^, heterogeneity only decreased from 90% to 64%, suggesting that there may be other causes contributing to the heterogeneity.

First, phase 2 and phase 3 trials were pooled together, despite phase 2 trials usually having smaller sample sizes, exploratory doses, different titration algorithms, and less strict monitoring, making them substantially less reliable. Nevertheless, given the meta-regression results, variability in achieved or protocol-driven dosing across trials should be considered a primary contributor to heterogeneity, with trial phase likely acting as a secondary source. This is supported by subgroup analysis, wherein heterogeneity for FBG in the insulin glargine subgroup was modest (*I*^2^ = 33%), having only pooled together phase 3 trials (Blevins, Philis-Tsimikas, Rosenstock)^[^13,17,18^]^, whereas the insulin degludec subgroup had high heterogeneity, consisting of two phase 2 and one phase 3 trial (Bue-Valleskey, Frias, Wysham)^[^16,19,20^]^. Moreover, the subgroups of insulin-naive and experienced patients both showed high heterogeneity, indicating that baseline insulin exposure is unlikely to explain the observed variability.

Further potential causes for heterogeneity include variation in follow-up duration. Since most trials used self-monitoring of blood glucose, frequency and adherence may vary (especially across regions with different health literacy), which may have contributed to outcome variability. FBS as an endpoint is inherently more variable than measures such as HbA1c, since it represents a single time-point measurement that is more susceptible to day-to-day fluctuations. However, the demonstrated dose–response relationship suggests that the null pooled effect for fasting blood glucose should be interpreted cautiously, as differences in dosing intensity and titration targets across studies may have partially masked true comparative effects. Importantly, a similar degree of heterogeneity has been reported in previous meta-analyses, suggesting that this variability may be intrinsic to the available evidence base rather than unique to the present study.

Our study found that efsitora was associated with an 11% increase in TEAEs. Tim Heise^[^22^]^, a crossover study that we did not include in our analysis for quality purposes, showed a higher incidence with efsitora (77.6%) compared to glargine (57.4%). It should be noted that these findings were mild and included symptoms such as headache and nasopharyngitis. In a previous meta-analysis^[^21^]^, treatment-emergent adverse effects were not commented upon.

In our pooled analysis, daily basal insulin was associated with a lower overall risk of TEAEs, with low heterogeneity. However, subgroup analyses indicated effect modification, as the increased risk with efsitora was mainly observed in insulin-experienced participants and in trials using insulin glargine, while no consistent excess risk was seen in insulin-naïve participants or with degludec comparators. This suggests that the overall safety signal may be driven by specific populations and comparator contexts rather than reflecting a uniform treatment effect.

Importantly, the excess events were largely systemic rather than local. The most frequently reported TEAEs across studies included nasopharyngitis, urinary tract infection, headache, diarrhea, arthralgia, back pain, dizziness, influenza, bronchitis, hypertension, anemia, and other mild infections, while local events such as injection-site reactions and administration errors occurred infrequently and at comparable rates between groups. This pattern indicates that the higher incidence of TEAEs with efsitora is not primarily driven by local tolerability issues related to injection volume or formulation, but rather by a slight increase in systemic events. Given the observed subgroup differences, these findings should be interpreted with caution and warrant further investigation in adequately powered trials to clarify whether patient population or comparator choice modifies the safety profile of weekly basal insulin therapy.

Hypoglycemia remains a pertinent concern with any insulin therapy. Overall, level 2 and 3 hypoglycemia did not differ meaningfully between groups; however, level 1 hypoglycemia showed effect modification depending on the comparator. Specifically, an increased risk was observed in trials using insulin degludec, while no significant difference was seen in trials using insulin glargine, reflecting a significant interaction. This suggests that the choice of comparator insulin may influence the incidence of mild hypoglycemia with efsitora, potentially due to differences in pharmacodynamic profiles or dosing strategies between daily and weekly basal insulins.

Our systematic review and meta-analysis found a 14% lower risk of nocturnal hypoglycemia with efsitora compared to daily insulin, consistent with Dutta^[^21^]^. Nocturnal hypoglycemia in diabetic patients has an incidence of 12–56%^[^23^]^, although it is likely underreported in routine practice. QWINT-3 and QWINT-4 RCTs reported higher rates of hypoglycemia during the first 12 weeks of efsitora treatment. This early increase may be explained by the loading dose needed to reach steady-state concentrations^[^21^]^. Overall, the reduction in nocturnal hypoglycemia may reflect an advantage of weekly-dosed insulin: daily basal insulin exhibits diurnal fluctuations, whereas weekly insulin provides a more stable pharmacokinetic profile^[^24^]^. Subgroup analyses indicated that the benefit in nocturnal hypoglycemia was mainly driven by insulin-experienced participants and trials using insulin glargine as the comparator. Clinically, efsitora would offer additional flexibility due to its 17-day half-life, implying that a delayed dose does not result in an immediate loss of efficacy^[^25^]^.

While our study presents many interesting findings concerning efsitora, many questions remain unanswered. Given the popularity of glucagon-like peptide-1 agonists, there should also be further research into combining weekly insulin with them. Such research is already being done in conjunction with once-daily basal insulin. Long-term safety data, particularly regarding cardiovascular outcomes, rare adverse events, and real-world adherence patterns, remain essential before widespread adoption. While the flexibility mentioned above may act as a potential benefit, it may also lead to patients becoming complacent and worsening their glycemic control.

From a clinical standpoint, these findings hold meaningful implications. Injection burden and treatment adherence remain persistent challenges in diabetes management. Once-weekly insulin offers a potentially transformative approach to improve adherence, reduce diabetes-related distress, and enhance patient satisfaction, particularly in populations with injection aversion or limited health literacy^[^26,27^]^. The comparable efficacy observed in this meta-analysis suggests that clinical adoption may not necessitate trade-offs in glycemic outcomes. However, safety outcomes need further exploration in high-quality trials.

Our study has several limitations. First, the number of available RCTs remains modest, with follow-up restricted to 1 year; long-term durability and safety of weekly insulin require further evaluation. Second, moderate-to-high statistical heterogeneity for certain outcomes underscores the need for cautious interpretation. Third, trial-level data precluded subgroup analyses by age, ethnicity, or comorbidities, which may influence treatment response.

Future research should explore real-world effectiveness, cost-effectiveness, and long-term safety, particularly regarding hypoglycemia, immunogenicity, and cardiovascular outcomes. Pragmatic trials and registry data will be crucial in confirming the generalizability of these findings.

## Conclusion

In conclusion, once-weekly insulin efsitora demonstrated glycemic efficacy and weight neutrality comparable to daily basal insulin analogs across six randomized controlled trials. These findings suggest that weekly insulin may represent a potential alternative to daily insulin that may alleviate treatment burden and enhance adherence without compromising metabolic control, although confirmation in broader settings is warranted. Additionally, efficacy outcomes, particularly fasting plasma glucose, should be interpreted with caution due to moderate heterogeneity across studies arising from differences in trial design, titration algorithms, and population characteristics. Safety outcomes were generally comparable between regimens, though a modest increase in TEAEs and a lower incidence of nocturnal hypoglycemia were noted, warranting continued post-marketing and long-term safety evaluation.

## Ethical approval

Not applicable.

## Consent

Not applicable.

## Data Availability

The data used to support the findings of this study are included within the article.
